# Supplementary material for: Implementation of Glucose-6-Phosphate Dehydrogenase (G6PD) testing for Plasmodium vivax case management, a mixed method study from Cambodia
Source: PLOS Glob Public Health. 2024 Jul 19;4(7):e0003476. doi: 10.1371/journal.pgph.0003476 (PMC11259306; doi:10.1371/journal.pgph.0003476)
Supplement: S1 Text — Examples of KII and FGD discussion guides. Discussion guides developed for health facility staff respondents and routine patient respondents. Table A in S1 Text. Variables collected from health facility and community health worker forms. Table B in S1 Text. Percent completeness of routine data gathered for analysis as compared to aggregated routine surveillance data. Table C in S1 Text. List of Key Informant Interviews, Focus Group Discussions, and Observations conducted. Table D in S1 Text. Demographic characteristics of KII and FGD participants. Table E in S1 Text. Plasmodium vivax episodes for which routine and non-routine care was received from nine selected health center catchment areas in Cambodia between January 2021 and March 2023 disaggregated by year. Table F in S1 Text. Initial point of care and referral rates from the community to health centers for G6PD testing and PQ treatment disaggregated by health center. Table G in S1 Text. Identified Advantages, Challenges, and Recommendations for the STANDARD G6PD Test (Biosensor). Table H in S1 Text. G6PD status of Plasmodium vivax patients tested for G6PD deficiency at nine health centers in Cambodia. Fig A in S1 Text. Median months between recurrences of patients presenting to health centers between January 2021 and March 2023. (PDF) [file pgph.0003476.s001.pdf]

Supplementary Information for

Implementation of Glucose-6-Phosphate Dehydrogenase (G6PD) testing for *Plasmodium vivax* case management, a mixed method assessment from Cambodia

Appendix A in S1 Text. Examples of KII and FGD discussion guides.

Table A in S1 Text. Variables collected from health facility and community health worker forms.

Table B in S1 Text. Percent completeness of routine data gathered for analysis as compared to aggregated routine surveillance data.

Table C in S1 Text. List of Key Informant Interviews, Focus Group Discussions, and Observations conducted.

Table D in S1 Text. Demographic characteristics of KII and FGD participants.

Table E in S1 Text. *Plasmodium vivax* episodes for which routine and non-routine care was received from nine selected health center catchment areas in Cambodia between January 2021 and March 2023 disaggregated by year.

Table F in S1 Text. Initial point of care and referral rates from the community to health centers for G6PD testing and PQ treatment disaggregated by health center.

Table G in S1 Text. Identified Advantages, Challenges, and Recommendations for the STANDARD G6PD Test (Biosensor).

Table H in S1 Text. G6PD status of *Plasmodium vivax* patients tested for G6PD deficiency at nine health centers in Cambodia.

Fig A in S1 Text. Median months between recurrences of patients presenting to health centers between January 2021 and March 2023.

Appendix A in S1 Text. Examples of KII and FGD discussion guides. Discussion guides developed for health facility staff respondents and routine patient respondents**.**

1. Discussion guide for health facility staff key informant interview 3.0
2. Discussion guide for routine patient key informant interview

*Discussion guides were tailored to each stakeholder type included in our study

**Not all data gathered from KIIs and FGDs using the discussion guides are presented in this study

DISCUSSION GUIDE FOR HEALTH FACILITY STAFF KEY INFORMANT INTERVIEW 3.0

Question	Information we are looking for
1. How long have you been working at this health facility?	**Length of employment at health facility.**
2. What does a usual workday look like for you?	**Daily work routine.** From getting to work to leaving to go home, what does the respondent usually do.
3. Is there malaria in this area?<br>a. Has it personally affected you? If so, how?<br>b. Do you treat many individuals with malaria?<br>c. What type of malaria have you treated the most?	**Malaria transmission in health facility area.** To get an idea of malaria transmission in the area.<br>Respondents' personal experiences with malaria. If they or their families have had it. Also, if they treat a lot of malaria, and if know what type of malaria (*P.v.*, *P.f.* or mixed).

QUANTITATIVE G6PD TESTING

4. How have vivax testing and treatment guidelines changed over time?	**A description of how vivax malaria testing and treatment guidelines have changed over time.**
5. Can you describe the diagnosis and treatment process of vivax patients at your health facility?<br>***Ask the participant to draw the workflow and describe.***<br>a. Have you heard of the G6PD enzyme?<br>b. Have you heard of G6PD deficiency?<br>c. Can you describe how this deficiency affects malaria treatment?<br>d. Is the process you describe what you do for all your vivax patients?	**Process of diagnosing vivax & administering radical cure + reasoning for G6PD testing.**<br><br>A detailed description of what is done to provide patients with radical cure (ASMQ +PQ) at health care providers (hospitals, health centres, VMWs) in Cambodia. The steps involved in diagnosis and treatment of vivax cases. Make sure they tell you by probing, if necessary, about G6PD status/testing, contraindications for PQ, follow-up.<br><br>→ also probe, if necessary, about their knowledge of the G6PD enzyme, G6PD

	deficiency, what it means for malaria i.e., why they need to do G6PD testing; the reason for this test.
6. How has your experience with the new treatment algorithm been?<br>a. What challenges have you faced in implementing this new testing and treatment algorithm?<br>[Probe] Have there been any challenges with regards to the procedures involved in testing patients for G6PD deficiency?	**Impressions, opinion, feeling about new treatment guidelines described before.** What they think of the new treatment guidelines/algorithm. Probe on specific **barriers/challenges to implementing the new test and treatment algorithm for vivax malaria.** This new includes malaria testing, quantitative G6PD testing, and administration of PQ if G6PD normal.
7. Is there something that could be improved to make the process easier?	**Recommendations and what could make the process of vivax diagnosis and treatment process easier.**
8. How has your experience with the Biosensor G6PD test been?<br>a. What do you think about the SD quantitative G6PD tests? [ease of use, integration into workflow, quality control, confidence in results]<br>b. What were your expectations? Has the test met them?	Description of their experience with the test. How they feel about it.<br><br>**Opinions about and expectations of the SD quantitative G6PD test**. Do they like and do they not like, and why. What do they not or do like about it. But by asking what you do you think, we are keeping it open to either positive or negative opinions about the test.<br>→ if not mentioned, probe about the cost of the test<br>→ the ease of use for all levels of the health system—especially for the health centre level. i.e., capacity/ability<br>→ and with the ease of use, the need for supervision for G6PD testing, the need for controls to make sure the test is functioning<br><br>Also, what they expected from the test and how it has turned out in reality. E.g., I thought that it would be a more reliable test, but now I can see,

	there is a lot of variation in the test results even from one person.
9. How confident do you feel in your ability to perform G6PD testing? On a scale of 1 not confident and 10 very confident.<br>a. Why do you feel this way?<br>b. What would make you feel more confident?	**Confidence of the health facility staff in using the test.** Are they confident and comfortable performing the test with an explanation of why they feel the way they do.
10. What do you expect will happen as a result of implementing quantitative G6PD testing?	**Expectations for the successful implementation of quantitative G6PD testing.** Objective of the implementing this new diagnostic → need for radical cure? (do not ask this outright, leave as open question and probe if necessary)
11. How have patients reacted to the G6PD testing process?	**Experience of patient reaction to G6PD testing process.** Based on the respondents' experience, how have patients reacted to the test.
12. Do you think VMWs should perform G6PD testing?<br>a. Why or why not?	**Opinion about the capacity of VMWs to perform G6PD testing and provide radical cure. + explanation of their opinions.**

VILLAGE MALARIA WORKER FOLLOW-UP

13. How have VMWs contributed to the vivax case management?	**Contribution of VMWs to vivax case management.** How VMWs have helped with the diagnosis and treatment of vivax malaria in Cambodia.
14. How has their role evolved? [should bring up VMW follow-up]<br>a. Can you describe the rationale behind VMW follow-up?<br>b. What do you expect from VMW follow-up?	**VMW role evolution.** Changes in their position, involvement, and what they do for malaria over time. → should bring up current VMW follow-up guidelines.<br>Make sure to probe on the reason VMW follow-up is needed and what is expected of it.

15. How do you see the role of VMWs in malaria case management in the future? [Probe for vivax specifically]	**Future for VMWs in malaria case management and control.** Future of having VMWs as part of malaria diagnosis and treatment, specifically vivax malaria. If they do not mention VMW follow-up for vivax, ask the reason why they did not mention it.
16. Can you describe what VMW follow-up entails?<br>a. [Probe] Can you describe your role in the VMW follow-up process?	**VMW follow-up process.** Describe the different activities that have to be done when conducting VMW follow-up.<br><br>Make sure to explore the r**ole of health facility staff in VMW-follow-up.** What do health facility staff do as part of the VMW-follow-up process. What are their responsibilities.
17. Is follow-up conducted for all vivax patients?<br>a. If not, why?	**Adherence to guidelines.** Whether VMWs are conducting VMW follow-up routinely. If not, reasons for which they are not doing it consistently.
18. What barriers has VMW follow-up faced?	**Barriers to VMW follow up.** Things that make it difficult to do or stop it from being done. What they are and if there is any way to solve the problems described.
19. Do you think VMW follow-up is helping with treatment adherence for vivax malaria?<br>a. If yes, how has it helped? And if not, why has it not helped?	**Effectiveness of VMW follow-up**. Has VMWs visiting patients made a difference in whether the patients take their medication and whether they relapse. Reasons behind their answers.
20. Would there be a better alternative than VMW follow-up to ensure patient adherence and safety?	**Alternative to VMW follow-up.**<br>Other options to make sure patients take their medications and are not experiencing serious adverse events.
21. Do you have any questions/ concerns or things that you would like us to know?	Anything else the respondent might want to say that they have not done so yet.
22. People we should be discussing with/learn from?	Any other person the respondent thinks we should talk to.

EFFORT

DAY 3 REVIEW VISIT

Thanks again for agreeing to meet with us again to further expand on some of the topics we discussed last time. Last time, we discussed the challenges involved with radical cure, especially around adherence to treatment and how VMW follow-up is meant to help with adherence and safety. That being the case, …

1. What would you think of the possibility of having a shorter stronger dose of the medicine? [Either having a single dose of TQ or a 7-day regimen.]	**Perceptions/Opinion on a shorter stronger dose of radical cure.** Do they think it would be useful, is it a good option or a bad one? Do not guide the question. Just ask a general open question and then also probe as to why they think what they think.
2. Have you heard of any shorter stronger doses of the medicine for vivax?	**Knowledge of the existence of shorter dose treatment options** e.g., 7-day PQ or 3-day PQ or 5-day PQ or single dose TQ.
3. What do you think would have to change if this stronger and shorter dose was used?<br>a. How might supervision change if the treatment for vivax malaria was a stronger, but shorter dose?	**Changes required to administer stronger and shorter doses.** Looking for changes in the diagnosis, treatment, follow-up/monitoring algorithm. Or any other changes they might think would be required.<br>→ should mention the need for more supervision/monitoring. If not, probe for it: **Changes in supervision/monitoring necessary for stronger but shorter treatment regimens**. For example: More or less monitoring, what type of monitoring, additional days of VMW follow-up. If not a follow-up interview make sure to establish what the current supervision/monitoring procedures are.
4. What would you think of the patient having to come back to the health facility for a review visit?<br>a. Would this visit be beneficial? Why or not?<br>b. If it is beneficial, how would it be?	**Perceptions and opinions about day three visit.** Do they think it is a bad or good idea, whether it is feasible, something patients would be okay doing etc. and their reasons for their opinions. Their experiences. Whether the visit would benefit the patients. And the reason why

	it may or may not have benefits—and what those benefits may be.
5. Would this visit help make sure patients take their medications? Why or not?	Opinions/perceptions based on experience about whether this visit would make sure/incentivize patients to take their treatment/medication. The reason for their opinion → share their experiences.
6. Would this visit help make sure patients are not experiencing adverse effects? Why or why not?	Opinions/perceptions based on experience about whether this visit would make sure patients are not experiencing side effects. The reason for their opinion → share their experiences.
7. What would or should happen during the day 3 visit?<br>a. Who would oversee *Pv* patient clinical reviews? Would one person be assigned for such work?	**Process and logistics that would be involved in the day-3 visit.** Steps involved, who would be involved, where. Workflow. What they would check for during the visit, how they would check for it.
8. How might this visit affect workflow?	**Day 3 visit impact on workflow.** How would having to conduct the day 3 visit medical exam impact the other work that needs to be done at the health facility. E.g., would it take away time from administrative tasks or seeing other patients or would it not impact very much…
9. How might adverse events or early warning signs be detected during the visit?	**Process of detecting adverse events or early warning signs.** What health facility staff will do detect adverse events or early warning signs. What health facility staff will do to make sure patients are not experiencing or exhibiting adverse events or early warning signs.
10. What would the response be to any adverse events or side effects of the drug?	**Response to adverse evets or drug side effects**. What will health facility staff do once they identify the adverse events or side effects. How will they mitigate them.
11. Would you feel confident/comfortable to conduct the clinical review?	**Confidence of health facility staff to conduct clinical review.** Would they feel okay doing the medical exam or do they think someone with more training should do it?

12. What operational challenges might exist in implementing a day 3 *Pv* patient visit?<br>a. How might a patient respond to having to come back to the health facility for further monitoring?<br>b. How might the health facility staff respond to conducting the medical exam?	**Barriers to or challenges involved with implementing this day 3 review visit.** Make sure to probe on receptivity or acceptance of this visit by patients, but also the receptivity and acceptance of health facility staff to conduct it.
13. a. What would make it easier for patients to take part in this day 3 visit?<br>b. What would make it easier for the health facility staff?<br>[probe--reduce workload for other activities, need of more staff, assign activities differently to the different staff?]	**Recommendations to make the day 3 visit easier for patients & health facility staff.** Other things that would be needed to implement the day three visit.
14. Might a VMW be assigned to a *Pv* patient to call or visit the patient to check up on them after their initial treatment?	**Role of VMW in Day 3 visit or monitoring.** Could the day 3 review visit be supplemented by VMW follow-up in day 7 and 14. What they think of this idea.
15. Do you have any questions/ concerns or things that you would like us to know?	Anything else the respondent might want to say that they have not done so yet.
16. People we should be discussing with/learn from?	Any other person the respondent thinks we should talk to.

EFFORT

DISCUSSION GUIDE FOR ROUTINE PATIENT KEY INFORMANT INTERVIEW

Introduction: We are interested in how you experience malaria and all steps in services related to malaria. Your experiences are important to improve care and treatment. There are no right or wrong answers. All information you tell us will not affect your treatment, and confidentiality and anonymity will be ensured. We will record this discussion so that we can write down and translate to English to let our team learn from your experience too. [Emphasize our independence].

Question	Information we are looking for
1. Have you had malaria before?<br>a. How many times?<br>b. How were you treated?<br>c. When?	**Malaria history.** Number of times, location of treatment, and when.
2. Do you know what type of malaria you have had?	**Previous malaria type.** E.g., vivax, falciparum, mixed…

QUANTITATIVE G6PD TESTING

3. Can you describe your most recent care-seeking (malaria diagnosis and treatment) experience?<br>a. How did you travel to get medical care?<br>b. Where were you diagnosed with malaria?<br>i. If you were diagnosed at the VMW level, were you referred to the district hospital or health centre?<br>c. When you arrived at the health facility did the physicians give you another test before treatment? [Probe G6PD testing]<br>i. What was your experience with G6PD testing at the health facility?<br>ii. How did you feel about giving your blood for testing?<br>iii. Do you know why they had to give you this test?	**Malaria care seeking experience.**<br><br>Detailed description of the last time the respondent had malaria from traveling to seek care to going home. Details of care location, process/steps (malaria testing, G6PD testing, treatment) → want them to tell a story with their feelings and answer these sub questions through their story. Not just focused on the process, the experience.<br><br>**G6PD experience and impressions and knowledge of reasons for the need of an extra test (G6PD testing).**

4. Were you given treatment after this test?<br>a. If yes, what treatment?<br>b. Can you describe what it was like taking the treatment?<br>i. Did you ever experience or hear about patients that got sick, like for example having blood in their urine, after getting treated with primaquine? What do you think about it?<br>c. What did the health facility staff tell you about the treatment?	**Treatment after test.** Treatment provided and experience taking the treatment and any counseling they received at the health facility.
5. Do you recognize this card? [*Show card for follow-up with G6PD status & Hb level]*<br>i. What do you think of when you see this card?<br>ii. Do you know why you are given this card?	**Recognition or knowledge of the Patient treatment and information form for Pv/mixed patient to track their treatment course.** Knowledge of why they were giving this card. Trying to get at the rationale of why it is important to take all the medicine. i.e. to prevent relapse.
6. Sometimes treatment can cause adverse events, including red/dark coloured urine, excessive fatigue, pale skin, shortness of breath. What happens if you have these symptoms?	**Knowledge of what happens/what to do if they experience adverse events or side effects.**
7. In the past, a few years ago, were you previously diagnosed with *P.v.* malaria? If you were, can you describe that went? *Probe: which experience they preferred and why*	**Description of experience of previous vivax malaria care-seeking experience.** Also, which treatment experience they preferred and why.

DRUG MONITORING: VMW FOLLOW-UP & DAY 3 CLINICAL REVIEW VISIT

8. When you last had malaria did you have any contact with doctors or health workers after you were given treatment at the health facility?	**Experience of treatment supervision or monitoring.** Doctor or health worker communicated with the respondent after initial treatment.
9. Did you have any support from anyone to complete your treatment?	**Whether the respondent received support for treatment completion.**
10. What kind of support did you receive? How often?<br>a. Describe what happens when the VMW comes to your house to check on you.<br>b. What did you do after you were given treatment?<br>i. Did you have to pay attention to anything in particular?<br>c. What did you think of the follow-up?<br>i. Has having this support from the VMW helped you?<br>ii. In what ways has it helped?<br>d. Is there something you would change about it?<br>e. Do you know why the VMW had to visit your house?	**Experience of support for treatment completion.** Probe for health worker support. Who provided the support and what kind of support it was. How often did they provide support.<br>→ should mention VMW follow-up. Then get a description of what happens when the VMW comes to visit the patients house.<br>→ Make sure get the role of the patient in the VMW follow-up process: what they had to pay attention to, what they had to fill out in terms of the form they are given to track their medication.<br>→ helpfulness of VMW follow-up: has it helped and how has it helped?<br>→ improvements to the process<br>→ knowledge of the reason for VMW follow-up.
11. What would happen if you had to go back to the health facility three days after being diagnosed and taking your first treatment dose? [Probe on challenges and barriers]	**Perceptions and opinions about day three visit.** Do they think it is a bad or good idea, whether it is feasible, something patients would be okay doing etc. and their reasons for their opinions. Their experiences.
12. Would you consider it useful? Why (not)? If yes, what would be the main use in your view [probe if better for adherence or safety]	Whether the visit would benefit the patients. And the reason why it may or may not have benefits—and what those benefits may be.

13. What would make it easier for you to take part in this day 3 visit?	**Recommendations to make the day 3 visit easier for patients**
14. How might this in person follow-up visit to the health facility compare to a VMW conducting follow-up?	**Comparison between VMW follow-up visit and day 3 medical review at the health facility.**
15. There are various types of radical cure regimen, what would you think of a shorter and stronger regimen?<br>a. How would this shorter dose be useful to you?	**Opinion** regarding a shorter but stronger dose of the medicine for vivax.
16. If having the day 3 visit meant having to take medicine only one time, would you be more open to it?	**Advantage of single dose treatment despite the need for a medical review at the health facility on day 3.**
17. Do you have any questions/ concerns or things that you would like us to know?	Anything else the respondent might want to say that they have not done so yet.
18. People we should be discussing with/learn from?	Any other person the respondent thinks we should talk to.

Table A in S1 Text. Variables collected from health facility and community health worker forms.

#	**List collected as part quantitative data collection**
1	Operational District
2	Health Center
3	Patient ID (MIS)
4	Form Source (HC/VMW)
5	Name
6	Sex
7	Age
8	Weight
9	Village Name
10	Contact Number
11	Point of Care
12	Malaria Type
13	Date
14	G6PD Test
15	G6PD Activity: UI/g Hb
16	Hb: g/dL
17	Counseling Completion
18	ACT
19	Radical Cure Treatment PQ
20	PQ Regimen
21	# of tablets
22	Follow Up Day 1 - Symptom Code
23	Follow Up Day 3 - Called
24	Follow Up Day 3 - Symptom Code
25	Follow Up Day 3 - Referred
26	Follow Up Day 7 - Called
27	Follow Up Day 7 - Symptom Code
28	Follow Up Day 7 - Referred
29	Follow Up Day 14 - Called
30	Follow Up Day 14 - Symptom Code
31	Follow Up Day 14 - PQ 7.5mg remaining
32	Follow Up Day 14 - Referred
33	Was this case referred to and/or treated by partner?
34	If yes, which partner?
35	Did health center record VMW follow-up?
36	Day 3 - VMW Follow-Up
37	Day 3 - VMW Symptom Code
38	Day 3 - VMW Referred
39	Day 7 - VMW Follow-Up
40	Day 7 - VMW Symptom Code
41	Day 7 - VMW Referred
42	Day 14 - VMW Follow-Up
43	Day 14 - VMW Symptom Code

44	Day 14 VMW - PQ 7.5mg remaining
45	Day 14 - VMW Referred
46	Day 3 - Partner Called
47	Day 3 - Partner Symptom Code
48	Day 3 - Partner Referred
49	Day 7 - Partner Called
50	Day 7 - Partner Symptom Code
51	Day 7 - Partner Referred
52	Day 14 - Partner Called
53	Day 14 - Partner Symptom Code
54	Day 14 - Partner PQ 7.5mg remaining
55	Day 14 - Partner Referred
56	Is patient recurrence and travel history available?
57	In the last 12 months, has the patient had vivax?
58	In the last 14 days, did the patient sleep in the village every night?
59	In the last 14 days, when the patient was not sleeping in their village, were they sleeping in Cambodia?
60	Village
61	Commune
62	District
63	Province
64	In the last 14 days, the patient sleep outside of Cambodia?
65	Which country did the patient sleep in?

Table B in S1 Text. Percent completeness of routine data gathered for analysis as compared to aggregated routine surveillance data.

Health Centre	Percent Completeness 2021	Percent Completeness 2022	Total
Chheu Tom	80%	100%	88%
Koh Preah	100%	100%	100%
Phnom Kravanh	88%	94%	91%
Pramoay	92%	92%	92%
Prognil	100%	98%	98%
Siem Pang	87%	99%	96%
Srae Kor	96%	100%	98%
Stueung Treng	0%	100%	98%
Svay Sor	100%	100%	100%

Table C in S1 Text. List of Key Informant Interviews, Focus Group Discussions, and Observations conducted.

Number	Study Site	Activity Description	Participant #
1	Phnom Kravanh	EFFORT Patient Pilot Interview 1- KII 1	1
2	Phnom Kravanh	EFFORT Patient Pilot Interview 2 - KII 2	1
3	Phnom Kravanh and Prongil	VMW Pilot Focus Group Discussion 1 (Biosensor Trained) - FGD 1	7
4	Phnom Kravanh	Referral Hospital Staff (Non-EFFORT) Pilot Focus Groups Discussion 2 - FGD 2	5
5	Phnom Kravanh	EFFORT Patient Pilot Interview 3 - KII 3	1
6	Phnom Kravanh	EFFORT Patient Pilot Interview 4 - KII 4	1
7	Pramoay	VMW Pilot Focus Group Discussion 3 - FGD 3	6
8	Pramoay	Routine Health Center Staff Pilot Interview 5 - KII 5	1
9	Phnom Kravanh	DHD Malaria Director Interview 6 - KII 6	1
10	Phnom Kravanh	EFFORT Lab Staff Interview 7 - KII 7	1
11	Phnom Kravanh	EFFORT Administrative Staff Interview 8 - KII 8	1
12	Phnom Kravanh	EFFORT/DH Doctor Interview 9 - KII 9	1
13	Phnom Kravanh	EFFORT Patient Interview 10 [Last day of follow-up] - KII 10	1
14	Phnom Kravanh	EFFORT Patient Interview 11 [Day 7] - KII 11	1
15	Phnom Kravanh	FGD with EFFORT/MORU staff 4 - FGD 4	6
16	Phnom Kravanh	EFFORT Patient Interview 12 [Day 3] - KII 12	1
17	Phnom Kravanh	EFFORT Patient Interview 13 [Day 3] - KII 13	1
18	Phnom Kravanh	EFFORT Patient Interview 14 [Day 3] - KII 14	1
19	Not Applicable	CNM Official Interview 15 - KII 15	1
20	Phnom Kravanh	EFFORT Patient Interviews [Day 3] [hybrid KII/FGD] 2 patients - KII/FGD U	2
21	Phnom Kravanh	EFFORT Patient Interview 16 [Day 3] - KII 16	1
22	Phnom Kravanh	EFFORT/DH Doctor - KII 17	1
23	Phnom Kravanh/Pramoay	EFFORT Patient Interview - KII 18	1
24	Phnom Kravanh/Pramoay	EFFORT Patient Inteview - KII 20	1
25	Phnom Kravanh	EFFORT Patient Inteview - KII 21	1
26	Pramoay	Routine VMW Interview - KII 19	1
27	Phnom Kravanh	EFFORT Patient Interview - KII 22	1
28	Phnom Kravanh	EFFORT Patient Interview - KII 23	1

29	Phnom Kravanh	EFFORT Patient Interview - KII 24	1
30	Pursat Provincial Health Dept	PHD Pursat Malaria Official Interview - KII 25	1
31	Phnom Kravanh	EFFORT Patient Interview - KII 26	1
32	Phnom Kravanh	Partner Interview - URC - KII 27	1
33	Phnom Kravanh	EFFORT/DH Doctor Interview - KII 28	1
34	Pursat Provincial Health Dept	PHD Pursat Malaria Official Interview (PMS) - KII 29	1
35	Chambak	Chambak HC Staff Interview - KII 30	1
36	Chambak	Chambak HC Staff Interview - KII 31	1
37	Chambak	VMW FGD 1 - FGD 5C	7
38	Chambak	MMW FGD 2 - FGD 6C	5
39	Chambak	Chambak HC Staff Interview - KII 32C	1
40	Chambak	Routine Patient Interview 1 (HC Chambak, June patient)- KII 33C	1
41	Chambak	Routine Patient Interview 2 (HC Chambak, July patient obv 2) - KII 34C	1
42	Chambak	Chambak HC Staff Interview - KII 35C	1
43	Chambak	Routine Patient Interview 3 (JHC Chambak, July patient obv 1 & 3) - KII 36C	1
44	Phnom Kravanh	Follow-Up with EFFORT Drs. (Bipin + Peakdey) - FGD U	1
45	Kampong Speu Provincial Health Dept.	Kampong Speu PHD Director Interview - KII 37C	1
46	Chambak	Routine Patient Interview 4 (HC Chambak, June patient) - KII 38C	1
47	Chambak	Routine Patient Interview 5 (HC Chambak, June patient) - KII 39C	1
48	Chambak	VMW Interview 1 - KII 40C	1
49	Kampong Speu Provincial Health Dept.	Kampong Speu Provincial Malaria Official - KII 41C	1
50	Chambak	HC Staff Chambak Follow-Up Interview - KII 31C Follow-Up	1
51	Phnom Srou Referral Hospital	CHAI Partner Interview 1 - KII 42C	1
52	Phnom Srou Referral Hospital	Phnom Srouch DHD/RH Malaria Stakeholders FGD - FGD 7C	6
53	Phnom Srou Referral Hospital	DHD Krokor Malaria Official Interview (OD Director) - KII 43K	1
54	Phnom Srou Referral Hospital	DHD Krokor Malaria Official Interview (ODMS) - KII 44K	1
55	Phnom Srou Referral Hospital	Routine Referral Hospital FGD - FGD 8	3
56	Chheu Tom	Chheu Tom HC Staff Interview 1 - KII 45K	1
57	Chheu Tom	Chheu Tom HC Staff Interview 2 - KII 46K	1

58	Chheu Tom	Chheu Tom HC Staff Interview 3 - KII 47K	1
59	Chheu Tom	Routine Patient Interview 6 (HC Chheu Tom, July patient) + wife - KII 48K	2
60	Chheu Tom	Routine Patient Interview 7 (HC Chheu Tom, July patient) + wife - KII 49K	2
61	Chheu Tom	VMW FGD Chheu Tom VMWs - FGD 9	7
62	Chheu Tom	Routine Patient Interview 8 (HC Chheu Tom, July patient) + wife - KII 50K	2
63	Chheu Tom	MMWs FGD Chheu Tom MMWs - FGD 10	4
64	Chheu Tom	Routine Patient Interview 9 (HC Chheu Tom, July patient) + wife - KII 51K	2
65	Chheu Tom	Routine Patient Interview 10 (HC Chheu Tom, July patient) - KII 52K	1
66	Chheu Tom	Chheu Tom HC Staff Interview 4 - KII 53K	1
67	Chheu Tom	Routine Patient Interview 11(HC Chheu Tom August Patient) - KII 54K	1
68	Chheu Tom	Routine Patient Interview 12 (HC Chheu Tom August Patient) - KII 55K	1
69	Siem Pang	Siem Pang EFFORT Study Staff Interview 1 (Dr.) - KII 56SP	1
70	Siem Pang	Siem Pang EFFORT Study Staff Focus Group Discussion - mini FGD	3
71	Not Applicable	CNM Official Interview - KII 57	1
72	Not Applicable	CNM Official Interview - KII 58	1
73	Not Applicable	CHAI Partner Central Interview - KII 59	1
74	Not Applicable	CNM Official Interview - KII 58 Follow-Up	1
75	Not Applicable	CNM Official Interview - KII 59 Follow-Up	1
76	Not Applicable	CNM Official Interview - KII 57 Follow-Up	1
77	Prongil	Day 3 Scenarios - FGD VMWs - Biosensor trained VMWs (Prongil HC) - D3E1	8
78	Prongil	Day 3 Scenarios - Patient Interview - D3E2	1
79	Phnom Kravanh	Day 3 Scenarios - Kravanh HC mini FGD - D3E3	3
80	Phnom Kravanh	Day 3 Scenarios - Kravanh Referral Hospital mini FGD - D3E4	3
81	Pramoay	Day 3 Scenarios - FGD VMWs - Routine (Pramoay) - D3E5	7
82	Pramoay	Day 3 Scenarios - Pramoay HC mini FGD - D3E6	3
83	Chheu Tom	Day 3 Scenarios - Patient mini-FGD - D3E7	3
84	Chheu Tom	Day 3 Scenarios - Chheu Tom HC mini FGD - D3E8	4
85	Not Applicable	Day 3 Scenarios - PMS - D3E9	-
86	Not Applicable	Day 3 Scenarios - CNM - D3E10	-
1	Phnom Kravanh	EFFORT Patient Day 3 Observation 1	-

2	Phnom Kravanh	EFFORT Patient Day 0 Observation 2	-
3	Phnom Kravanh	EFFORT Patient Day 0 Observation 3	-
4	Phnom Kravanh	EFFORT Patient Day 0 Observation 4	-
5	Phnom Kravanh	EFFORT Patient Day 3 Observation 2	-
6	Phnom Kravanh	EFFORT Patient Day 3 Observation 3	-
7	Phnom Kravanh	EFFORT Patient Day 3 Observation 4	-
8	Phnom Kravanh	EFFORT Patient Day 0 Observation 5	-
9	Phnom Kravanh	EFFORT Patient Day 3 Observation 5	-
10	Phnom Kravanh	EFFORT Patient Day 3 Observation 6	-
11	Phnom Kravanh	EFFORT Patient Day 3 Observation 7R	-
12	Phnom Kravanh	EFFORT Patient Day 0 Observation 6	-
13	Phnom Kravanh	EFFORT Patient Day 0 Observation 7	-
14	Phnom Kravanh	EFFORT Patient Day 3 Observation 8	-
15	Phnom Kravanh	EFFORT Patient Day 3 Observation 9	-
16	Phnom Kravanh	EFFORT Patient Day 3 Observation 10	-
17	Phnom Kravanh	EFFORT Patient Day 3 Observation 11	-
18	Phnom Kravanh	EFFORT Patient Day 3 Observation 12	-
19	Phnom Kravanh	EFFORT Patient Day 3 Observation 13	-
20	Phnom Kravanh	EFFORT Patient Day 3 Observation 14	-
21	Phnom Kravanh	EFFORT Patient Day 3 Observation 15	-
22	Chambak	Routine Patient Diagnosis & Treatment Observation 1 (RDT negative)	-
23	Chambak	Routine Patient Diagnosis & Treatment Observation 2 (VMW referred)	-
24	Chambak	Routine Patient Diagnosis & Treatment Observation 3 (Former RDT negative 1)	-
25	Chheu Tom	Routine Patient Diagnosis & Treatment Observation 4 (Microscopy Negative)	-
26	Chheu Tom	Routine Patient Diagnosis & Treatment Observation 5 (RDT+Microscopy Negative)	-
27	Chheu Tom	Routine Patient Diagnosis & Treatment Observation 6 (Microscopy Negative)	-
28	Chheu Tom	Routine Patient Diagnosis & Treatment Observation 7 (RDT Positive)	-
29	Chheu Tom	Routine Patient Diagnosis & Treatment Observation 8 (Pm)	-
30	Chheu Tom	Routine Patient Diagnosis & Treatment Observation 9 (VMW referred)	-
31	Chheu Tom	Routine Patient Diagnosis & Treatment Observation 10 (Microscopy Negative)	-

Table D in S1 Text. Demographic characteristics of KII and FGD participants.

Participant Number	Discussion Type	Discussion Number	Respondent Number (FGD)	Manuscript Code	Age	Gender	Occupation	Follow-up
1	Interview	1	Interview	pp1	57	Male	EFFORT Patient - Teacher	No
2	Interview	2	Interview	pp2	27	Male	EFFORT Patient - Farmer	No
3	Focus Group Discussion	1	R1	chw1	51	Male	VMW (Farmer)	No
4	Focus Group Discussion	1	R2	chw2	39	Female	VMW (Groceries seller)	No
5	Focus Group Discussion	1	R3	chw3	50	Female	VMW (Farmer)	No
6	Focus Group Discussion	1	R4	chw4	24	Male	VMW (Farmer)	No
7	Focus Group Discussion	1	R5	chw5	41	Female	VMW (Farmer)	No
8	Focus Group Discussion	1	R6	chw6	49	Female	VMW (Farmer)	No
9	Focus Group Discussion	1	R7	chw7	56	Female	VMW (Farmer)	No
10	Focus Group Discussion	2	R1	rh1	29	Female	Referral Hospital Nurse	Yes
11	Focus Group Discussion	2	R2	rh2	27	Female	Referral Hospital Nurse	Yes
12	Focus Group Discussion	2	R3	rh3	29	Male	Referral Hospital Nurse	Yes
13	Focus Group Discussion	2	R4	rh4	26	Female	Referral Hospital Nurse	No
14	Focus Group Discussion	2	R5	rh5	30	Male	Referral Hospital Nurse	No
15	Interview	3	Interview	pp3	30	Female	Farmer (former VMW)	No
16	Interview	4	Interview	pp4	22	Male	Farmer	No
17	Focus Group Discussion	3	R1	chw8	35	Female	VMW (Housewife)	Yes

18	Focus Group Discussion	3	R2	chw9	63	Male	VMW (Farmer)	No
19	Focus Group Discussion	3	R3	chw10	33	Male	VMW (Farmer)	No
20	Focus Group Discussion	3	R4	chw11	61	Male	VMW (Farmer)	Yes
21	Focus Group Discussion	3	R5	chw12	41	Female	VMW (Groceries seller)	Yes
22	Focus Group Discussion	3	R6	chw13	62	Female	VMW (Groceries seller)	Yes
23	Interview	5	Interview	hc1	45	Male	Health Centre Nurse	Yes
24	Interview	6	Interview	do1	50	Male	Operational District Malaria Supervisor	No
25	Interview	7	Interview	etl1	27	Female	EFFORT Lab Staff	Yes
26	Interview	8	Interview	et1	34	Male	EFFORT Assistant	No
27	Interview	9	Interview	etd1	34	Female	Referral Hospital Doctor and EFFORT Doctor	Yes
28	Interview	10	Interview	pp6	47	Male	EFFORT Patient - Environment officer	No
29	Interview	11	Interview	pp7	18	Male	EFFORT Patient - Farmer	No
30	Focus Group Discussion	4	R1	et2	34	Male	EFFORT staff	No
31	Focus Group Discussion	4	R2	et3	31	Male	Referral Hospital Nurse and EFFORT Nurse	No
32	Focus Group Discussion	4	R3	et4	28	Female	Referral Hospital Nurse and EFFORT Nurse	No
33	Focus Group Discussion	4	R4	et5	24	Female	Referral Hospital Nurse and EFFORT Nurse	No
34	Focus Group Discussion	4	R5	etl2	29	Female	EFFORT Lab Staff	No
35	Focus Group Discussion	4	R6	etl3	27	Male	EFFORT Lab Staff	Yes
36	Interview	12	Interview	pp8	31	Male	EFFORT Patient - Construction worker	No
37	Interview	13	Interview	pp9	50	Male	EFFORT Patient - Farmer	No

38	Interview	14	Interview	pp10	18	Male	EFFORT Patient - Labor worker	No
39	Interview	15	Interview	cnm1		Male	National Malaria Program/CNM Official	No
40	Interview	15a	R1	pp11	37	Male	EFFORT Patient - Farmer	No
41	Interview	15a	R2	pp12	18	Male	EFFORT Patient - Farmer	No
42	Interview	16	Interview	pp13	18	Male	EFFORT Patient - Farmer	No
43	Interview	17	Interview	etd2	30	Female	Referral Hospital Doctor and EFFORT Doctor	Yes
44	Interview	18	Interview	pp14	25	Female	EFFORT Patient - Farmer	No
45	Interview	19	Interview	chw14	25	Female	VMW (Online Seller)	No
46	Interview	20	Interview	pp15	18	Male	EFFORT Patient - Farmer	No
47	Interview	21	Interview	pp16	34	Male	EFFORT Patient - Farmer	No
48	Interview	22	Interview	pp17	36	Male	EFFORT Patient - Labor Worker	No
49	Interview	23	Interview	pp18	18	Male	EFFORT Patient - Farmer	No
50	Interview	24	Interview	pp19	47	Female	EFFORT Patient - Farmer	No
51	Interview	25	Interview	po1	50	Male	Provincial Health Department Official	No
52	Interview	26	Interview	pp20	39	Female	EFFORT Patient - Farmer	No
53	Interview	27	Interview	cnmp1	50	Male	CNM Partner (URC staff)	No
54	Interview	28	Interview	etd3	36	Male	Referral Hospital Doctor and EFFORT Doctor	Yes
55	Interview	29	Interview	po2	50	Female	Provincial Malaria Supervisor	No
56	Interview	30C	Interview	hc2	55	Male	Health Centre Clerk	No
57	Interview	31C	Interview	hc3		Female	Health Centre Nurse	Yes
58	Focus Group Discussion	5C	R1	chw15	51	Female	VMW (Farmer)	No
59	Focus Group Discussion	5C	R2	chw16	54	Female	VMW (Farmer)	No
60	Focus Group Discussion	5C	R3	chw17	65	Male	VMW (Farmer)	No
61	Focus Group Discussion	5C	R4	chw18	63	Male	VMW (Farmer)	No

62	Focus Group Discussion	5C	R5	chw19	43	Male	VMW (Farmer)	No
63	Focus Group Discussion	5C	R6	chw20	66	Male	VMW (Farmer)	No
64	Focus Group Discussion	5C	R7	chw21	52	Male	VMW (Farmer)	No
65	Focus Group Discussion	6C	R1	chw22	52	Male	VMW (Farmer)	No
66	Focus Group Discussion	6C	R2	chw23	58	Male	VMW (Farmer)	No
67	Focus Group Discussion	6C	R3	chw24	58	Male	VMW (Farmer)	No
68	Focus Group Discussion	6C	R4	chw25	55	Male	VMW (Farmer)	No
69	Focus Group Discussion	6C	R5	chw26	45	Female	VMW (Farmer)	No
70	Interview	32C	Interview	hc4	26	Female	Midwife	No
71	Interview	33C	Interview	pp20	54	Male	Routine patient - School Director	No
72	Interview	34C	Interview	pp21	18	Male	Routine patient - Find Forest Products	No
73	Interview	35C	Interview	hc5	33	Male	Health Center Director's Assistant - Malaria Controller	No
74	Interview	36C	Interview	pp22	33	Male	Routine patient - Wild Aid staff	No
75	Interview	37C	Interview	po3	50	Male	Provincial Health Department Official	No
76	Interview	38C	Interview	pp23	29	Female	Routine patient - Corn vender (Farmer)	No
77	Interview	39C	Interview	pp24	24	Female	Routine patient - Find forest products	No
78	Interview	40C	Interview	chw27	54	Male	VMW (Farmer)	No
79	Interview	41C	Interview	po4	28	Female	Provincial Malaria Supervisor	Yes
80	Interview	42C	Interview	cnmp2	34	Male	Operational District Partner	No
81	Focus Group Discussion	7C	R1	do2	33	Male	Operational District Official	No
82	Focus Group Discussion	7C	R2	rh6	50	Female	Referral Hospital Assistant	No

83	Focus Group Discussion	7C	R3	do3	29	Male	Operational District Malaria Supervisor	No
84	Focus Group Discussion	7C	R4	rh7	57	Male	Referral Hospital Director	No
85	Focus Group Discussion	7C	R5	rhd1	55	Male	Referral Hospital Doctor	No
86	Focus Group Discussion	7C	R6	rhl1	30	Male	Referral Hospital Lab Staff	No
87	Interview	43K	Interview	do4	70	Male	Operational District Official	No
88	Interview	44K	Interview	do5	31	Male	Operational District Malaria Supervisor	No
89	Focus Group Discussion	8	R1	rhl2	54	Male	Referral Hospital Lab Staff	No
90	Focus Group Discussion	8	R2	rh8	32	Male	Referral Hospital Nurse	No
91	Focus Group Discussion	8	R3	rh9	34	Female	Referral Hospital Nurse	No
92	Interview	45K	Interview	hc6	54	Female	Lab Staff + Private Clinic Owner	Yes
93	Interview	46K	Interview	hc7	53	Male	Consultations Doctor + Private Clinic Owner	Yes
94	Interview	47K	Interview	hc8	36	Female	Midwife + Private Clinic Owner	Yes
95	Interview	48K	Interview	ps1	20	Female	Routine patient spouse- Farmer	No
96	Interview	48K	Interview	pp25	25	Male	Routine patient - Find Forest Products	No
97	Interview	49K	Interview	pp26	27	Male	Routine patient - Find Forest Products	No
98	Interview	49k	Interview	ps2	36	Female	Routine patient spouse - Groceries Seller	No
99	Focus Group Discussion	9	R1	chw28	64	Male	VMW (Farmer)	No
100	Focus Group Discussion	9	R2	chw29	37	Female	VMW (Farmer)	No
101	Focus Group Discussion	9	R3	chw30	61	Male	VMW (Farmer)	No
102	Focus Group Discussion	9	R4	chw31	43	Male	VMW (Farmer)	No

103	Focus Group Discussion	9	R5	chw32	44	Male	VMW (Farmer)	No
104	Focus Group Discussion	9	R6	chw33	57	Male	VMW (Farmer)	No
105	Focus Group Discussion	9	R7	chw34	28	Female	VMW (Farmer)	No
106	Interview	50K	Interview	pp27	36	Male	Routine patient - Find Forest Products	No
107	Interview	50K	Interview	ps3	39	Female	Routine patient spouse -Farmer	No
108	Focus Group Discussion	10	R1	chw35	42	Female	MMW (Farmer)	No
109	Focus Group Discussion	10	R2	chw36	55	Female	MMW (Farmer)	No
110	Focus Group Discussion	10	R3	chw37	29	Female	MMW (Groceries Seller)	No
111	Focus Group Discussion	10	R4	chw38	30	Female	MMW (Farmer)	No
112	Interview	51K	Interview	pp28	30	Male	Routine patient -Find Forest Product (Farmer)	No
113	Interview	51K	Interview	ps4	27	Female	Routine patient spouse - Groceries Seller	No
114	Interview	52K	Interview	pp29	19	Male	Routine patient - Find Forest Products	No
115	Interview	53K	Interview	hc9	55	Male	Health Centre Nurse - Malaria Controller	Yes
116	Interview	54k	Interview	pp30	45	Male	Routine patient - Forestry Administrations	No
117	Interview	55K	Interview	pp31	68	Male	Routine patient - Forestry Administrations	No
118	Interview	56SP	Interview	etd4	57	Male	EFFORT Doctor	No
119	Focus Group Discussion	11SP	R1	et6	51	Female	Coordinator (Nurse)	No
120	Focus Group Discussion	11SP	R2	et7	31	Female	EFFORT Nurse	No
121	Focus Group Discussion	11SP	R3	etl4	30	Male	EFFORT Lab Staff	No
122	Interview	57	Interview	cnm2		Male	CNM Technical Director	Yes

123	Interview	58	Interview	cnm3		Male	CNM Deputy Director	Yes
124	Interview	59	Interview	cnmp3		Male	CHAI Central Level Official	Yes
125	Focus Group Discussion	D3E1 - 13	R1	chw39	71	Male	VMW (Farmer)	No
126	Focus Group Discussion	D3E1 - 13	R2	chw40	31	Female	VMW (Farmer)	No
127	Focus Group Discussion	D3E1 - 13	R3	chw41	40	Female	VMW (Farmer)	No
128	Focus Group Discussion	D3E1 - 13	R4	chw42	41	Female	VMW (Farmer)	No
129	Focus Group Discussion	D3E1 - 13	R5	chw43	26	Female	VMW (Farmer)	No
130	Focus Group Discussion	D3E1 - 13	R6	chw44	61	Female	VMW (Farmer)	No
131	Focus Group Discussion	D3E1 - 13	R7	chw45	56	Female	VMW (Farmer)	No
132	Focus Group Discussion	D3E1 - 13	R8	chw46	44	Female	VMW (Farmer)	No
133	Interview	D3E2 - 60	Interview	pp32	45	Male	Routine patient - Farmer	No
134	Focus Group Discussion	D3E3 - 14	R1	hc10	45	Male	Health Centre Lab Staff	No
135	Focus Group Discussion	D3E3 - 14	R3	hc11	50	Male	Health Centre Nurse - Malaria Controller	No
136	Focus Group Discussion	D3E5 - 16	R2	chw47	34	Female	VMW (Farmer)	No
137	Focus Group Discussion	D3E5 - 16	R3	chw48	56	Female	VMW (VHSG)	No
138	Focus Group Discussion	D3E5 - 16	R7	chw49	23	Female	VMW (Farmer)	No
139	Focus Group Discussion	D3E6- 17	R1	hc12	29	Male	Health Centre Lab Staff	No
140	Focus Group Discussion	D3E7 - 18	R1	pp33	32	Male	Farmer	No
141	Focus Group Discussion	D3E7 - 18	R2	pp34	27	Male	Farmer	No

142	Focus Group Discussion	D3E7 - 18	R3	pp35	20	Male	Farmer	No

Table E in S1 Text. *Plasmodium vivax* episodes for which routine and non-routine care was received from nine selected health center catchment areas in Cambodia between January 2021 and March 2023 disaggregated by year.

Health center	Number of vivax episodes											
	2021			2022			Jan-Mar 2023			Total		
	Routine	Non-Routine	Total	Routine	Non-Routine	Total	Routine	Non-Routine	Total	Routine	Non-Routine	Total
Chheu Tom	96	7	103	85	8	93	7	0	7	188	15	203
Phnom Kravanh	59	33	92	35	33	68	3	0	3	97	66	163
Pramoay	60	36	96	34	15	49	5	0	5	99	51	150
Prongil	12	11	23	14	6	20	0	0	0	26	17	43
Svay Sor	12	0	12	12	1	13	0	0	0	24	1	25
Koh Preah	17	0	17	58	0	58	40	0	40	115	0	115
Siem Pang	191	25	216	397	141	538	82	0	82	670	166	836
Srae Kor	51	0	51	26	0	26	1	0	1	78	0	78
Steung Traeng	0	0	0	45	0	45	3	0	3	48	0	48
Total	498	112	610	706	204	910	141	0	141	1,345	316	1,661

Table F in S1 Text. Initial point of care and referral rates from the community to health centers for G6PD testing and PQ treatment disaggregated by health center.

Health Centre	Total	HC	CHW Cases	Unknown	CHW cases not reached	% CHW	% not reached HC	% reached	Median distance between village and HC	Majority Patient Type
Svay Sor	25	3	22	0	0	88.0%	0.0%	100.0%	4.5 km	Villagers – day laborer or local farmers
Prognil	43	19	24	0	1	55.8%	4.2%	95.8%	5 km	Villagers – day laborer or local farmers
Pramoay	150	51	99	0	11	66.0%	11.1%	88.9%	14 km	Villagers – day laborer or local farmers and forest goers
Chheu Tom	203	35	168	0	22	82.8%	13.1%	86.9%	4 km	Villagers – day laborer or local farmers and forest goers
Phnom Kravanh	163	74	74	15	13	45.4%	17.6%	82.4%	3.5 km	Villagers – day laborer or local farmers and forest goers
Siem Pang	836	65	747	24	516	89.4%	69.1%	30.9%	9.5 km	Forest-goers, mobile-migrant, military
Koh Preah	115	75	40	0	36	34.8%	90.0%	10.0%	17 km	Villagers, Forest-goers
Srae Kor	78	69	9	0	9	11.5%	100.0%	0.0%	29 km	Villagers, Forest-goers
Stung Treng	48	48	0	0	0	0.0%	NA	NA	6 km	Urban town residents

Table G in S1 Text. Identified Advantages, Challenges, and Recommendations for the STANDARD G6PD Test (Biosensor).

Identified Advantages of STANDARD G6PD Test

Device	Device Results		Device + Complete Treatment
Small and portable<br>Easy to use<br>Fast (2 mins)<br>G6PD **and** Haemoglobin level<br>Numeric output - easy interpretation<br>Provides date & time setting<br>Acceptable enough test results<br>Small % of invalid test - few errors<br>Faster treatment process<br>Allowing for women/intermediates to (safely) receive treatment<br>Availability of control raegents	Health literacy	Device purpose - perceived advantage:<br>**Safe** complete treatment - differential treatment at PoC	**Less** recurrence - reduction in cases<br>Reduction in personal economic burden<br>Malaria elimination

Identified Challenges of STANDARD G6PD Test

Program

Training	Monitoring	Supply	Central Level
• Frequency<br>• Model (on the job vs formal training)<br>• Integration of training in broader case management (budgetary constraints)	• Budget<br>• Frequency<br>• Integration	• No WHO pre-qualification<br>• Test strips shelf-life<br>• Quarterly National Distribution<br>• Test strip package size (cost-effectiveness)	• Lack of trust in G6PD test haemoglobin reading<br>• Lack of emphasis of importance of radical cure at central level in trainings and job aids

Point of Care

End-users	G6PD Test Device	Patient
• Training / confidence in testing ability<br>• Lack of practice<br>• Lack of trust in G6PD test results<br>• Communicating radical cure as magic bullet for vivax malaria<br>• Fear of drug-induced haemolysis<br>• Lack of trained staff<br>• Care of device<br><br>Affecting test results:<br>• Blood collection (finger prick, breaking RBC)<br>• Time btw blood collection & testing<br>• Alcohol drying prior to blood collection	• Device and test strip care and storage<br>• Wait time after refrigeration<br>• Difficult control process<br>• Relatively complex testing process<br>• Inconsistency in test results<br>◦ Mixing blood with buffer<br>◦ Amount of blood<br>◦ Knowing which test results to trust<br>• Complex testing process<br>-> Test errors<br>◦ Code chip matching<br>◦ Pipetting<br>◦ Order of steps<br>◦ Leaving flap open too long<br>◦ Storage of test strips<br>◦ Low battery<br>◦ Battery quality	• Fear of pain finger prick (multiple fingerpricks)<br>• Fear of loss of blood (veinous blood draw)<br>• Cost of transport to HC<br>• Opportunity cost traveling to HC<br>• Distance to HC<br>• Road conditions<br>• Perception of the severity of disease<br>• Understanding of vivax treatment<br>◦ Impact on work<br>◦ Normal treatment = 3 days<br><br>**IEC/BCC**<br>• Counselling of patients around complete treatment (e.g. quality & content)

Identified Recommendation for STANDARD G6PD Test		
**G6PD Test Device**<br>**Device**<br>• Chargeable test device not reliant on single use batteries.<br>• Stronger/hard plastic storage box for test device (analyzer).<br><br>**Process simplification**<br>• Having the same chip code number.<br>• Removing the cover of the sample well (order of steps).<br>• Increasing size of sample well.<br>• No refrigeration required or waiting after taking out from refrigeration.<br>• Make it like a malaria RDT or glucose test - mixing step with buffer not required.<br>• Reducing Ezi Tube use from two to one.<br>• Ezi Tube that only allows for the collection of required amount.<br>• Having all in one malaria and G6PD test (all tests related to malaria case management).<br><br>**Interpretation simplification**<br>• G6PD device that produces test interpretation for treatment (treatment recommendation)	**End-users**<br>**Blood collection**<br>• Doing a veinous blood draw when multiple finger pricks would be required.	**Program**<br>**Incentives**<br>• Providing incentives for patients to come to HC for G6PD testing<br><br>**Workflow**<br>• At RH level, place G6PD test in treatment area + training doctors and nurses to use the machine (vs laboratory staff)<br><br>**Tranings**<br>• More frequent trainings

Table H in S1 Text. G6PD status of *Plasmodium vivax* patients tested for G6PD deficiency at nine health centers in Cambodia.

**G6PD tested**	Tested as G6PD normal*	Tested as G6PD intermediate**	Tested as G6PD deficient***	No G6PD results available	Total tested
Number of males	483 (61%)	NA (0%)	206 (26%)	2 (0.25%)	691(87%)
Number of females	58 (7%)	23 (3%)	16 (2%)	2 (0.25%)	99 (13%)
Total tested	541 (68%)	23 (3%)	222 (28%)	4 (0.5%)	790 (100%)

*Per STANDARD G6PD test results defined as:
- 6 U/g Hb or over for females
- 6 U/g Hb or over for males between February 2021 and May 2022
- 4 U/g Hb or over for males between June 2022 and March 2023

** Per STANDARD G6PD test results defined as:
- Females between 4 and 6 U/g Hb

*** Per STANDARD G6PD test results defined as:
- Under 6 U/g Hb for females
- Under 6 U/g Hb for males between February 2021 and May 2022
- Under 4 U/g Hb for males between June 2022 and March 20233

Fig A in S1 Text. Median months between recurrences of patients presenting to health centers between January 2021 and March 2023.

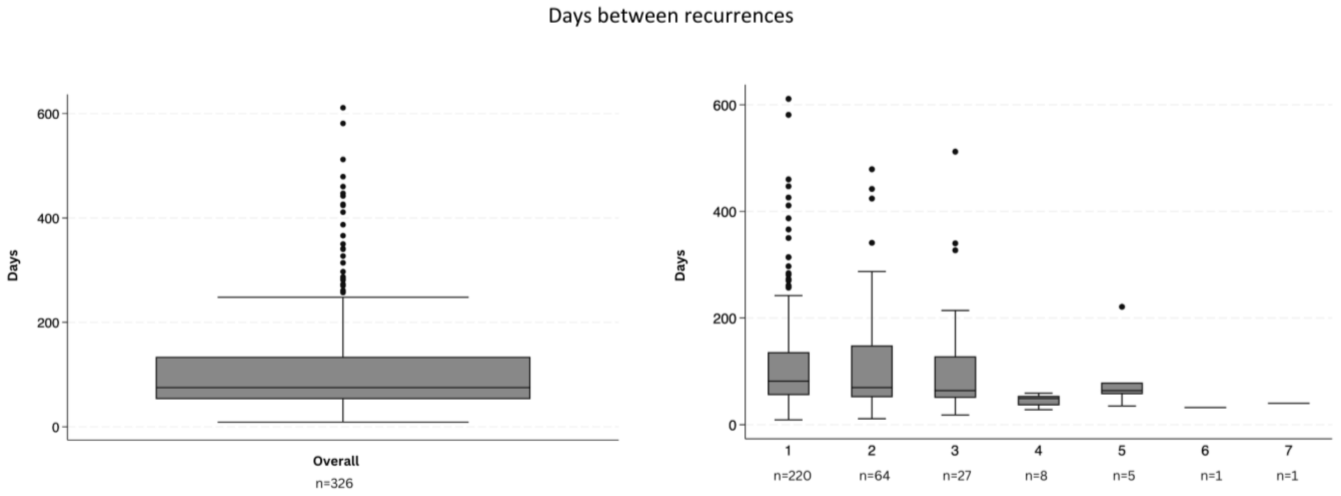

Overall = overall time between vivax cases
1 = time between 1st and 2nd vivax case
2 = time between 2nd and 3rd vivax case
3 = time between 3rd and 4th vivax case
4 = time between 4th and 5th vivax case
5= time between 5th and 6th vivax case
6 = time between 6th and 7th vivax case
7 = time between 7th and 8th vivax case
