# Supplementary material for: Implementation of Glucose-6-Phosphate Dehydrogenase (G6PD) testing for Plasmodium vivax case management, a mixed method study from Cambodia
Source: PLOS Glob Public Health. 2024 Jul 19;4(7):e0003476. doi: 10.1371/journal.pgph.0003476 (PMC11259306; doi:10.1371/journal.pgph.0003476)
Supplement: Attachment — Submitted filename: G6PD Implementation_Rebuttal Letter.pdf [file pgph.0003476.s002.pdf]

menzies
school of health research
CHARLES DARWIN UNIVERSITY AUSTRALIA
Postal: PO Box 41096, Casuarina NT 0811, Australia
Location: John Mathews Building (Bldg 58),
Royal Darwin Hospital Campus, Rocklands Drv, Casuarina NT 0810
Ph: (08) 8946 8600 Fax: (08) 8946 8464
Website: menzies.edu.au
ABN: 70 413 542 847

Darwin, June 17, 2024

Dear Editor,

Thank you to you and the reviewers for taking the time to review our manuscript and providing useful feedback. We have carefully reviewed the feedback and made the necessary changes to the manuscript.

With regards to the editorial journal requirements, we have reviewed the references list to ensure it is complete and correct and removed any copyright or trademark symbols from the manuscript. We have also included PLOS' questionnaire on inclusivity in global research as part of our revised submission. Please find replies to each comment from reviewers below.

**Reviewer 1:**

**Comment 1:** The manuscript on implementation of G6PD testing in Cambodia uses mixed methods approach to address the use of G6PD tests. Half of *P. vivax* diagnosis in the field are able to reach health centers to obtain testing for G6PD. Cambodia has 4,000 cases of malaria with about 90% P. vivax. Please note in introduction or discussion that those with only 10% (deficient) of normal levels are at most risk of hemolysis requiring change in dosing of primaquine to one per week for 8 weeks and no dosing of tafenoquine. While this in figure 3, it needs to be stated.

**Reply**: A sentence clause addressing the treatment recommendation for G6PD deficient vivax malaria patients was added to the introduction on line 102 (page 4) of the clean version of the document. The sentence was changed to, "Primaquine, at a low dose of 3.5mg/kg, has been recommended in the Cambodian National Treatment Guidelines since 2014 as a treatment for vivax malaria in patients with confirmed G6PD normal status, *while deficient individuals were not to receive primaquine* (15–17). *Despite the recommendation for G6PD normal individuals*, patients were not treated with primaquine because of a relatively high prevalence of G6PD deficiency of 8-19% (18–21), a lack of PoC G6PD testing (15), and concerns about severe hemolysis following drug exposure (15)." Treatment recommendations are explained in more detail in the "Overview of routine vivax case management policy in Cambodia" sub-section of the methods section.

**Comment 2**: Explain how 437 P. vivax patients make it to the clinic yet 790 or 353 more are tested in abstract. Abstract should note that most of these had both diagnosis and G6PD testing in Health center. What is the denominator for the 353 G6PD tests that did not originate from field. The approximate numbers come out later in results abut need clarification in the abstract. Where is the 353 in the flow chart of numbers?

**Reply**: We clarified that the 790 patients tested consisted of eligible patients who presented directly to the health center (360/407) and those that were initially diagnosed by CHWs (429/434). The sentence from line 58 to 60 (page 2) now reads: "93.9% (790/841) of eligible vivax malaria patients *who successfully completed referral (429/434) and directly presented to the health center (360/407)* were tested with the Biosensor."

Reviewer 1 also suggested that the disaggregation by initial point of care for G6PD testing be included in Figure 3 of the manuscript. Figure 3 presents aggregated G6PD testing and treatment data (regardless of initial point of care) once at the health center for clarity and simplification of the figure. The figure indicates that 868 patients were screened for G6PD testing at the health center, 437 of whom were successfully referred to the health center from the community and 430 of whom presented directly to the health center. Therefore, for clarity of the figure this disaggregation was not added.

**Comment 3:** The perceptions are important of the utility or cost benefit for taking the 14 days of meds. The low use in females and those under 20kg is important enough to move to abstract.

**Reply**: We added treatment related findings to the abstract on lines 62-64 (page 2): "*Of the eligible 1,213 vivax episodes, only 443 (36.5%) were appropriately treated with primaquine. 70.5% (165/234) of female patients and all children under 20 kilograms never received primaquine*." As a result of the two suggested additions to the abstract, the word count is now slightly over the 300-word limit. We leave it to the editor to advise if this is acceptable.

**Comment 4:** The authors should comment on a possible monthly plan to carry G6PD tests to villages to test and treat with primaquine those unable to make the journey to health center. I know there are refrigeration issues, but these might be overcome. The timing of primaquine is not important after treatment with the blood stage treatment also providing post treatment prophylaxis from new blood stages for a few weeks**.**

**Reply:** Thank you for your suggestion and thoughts on alternative ways to bring G6PD testing and primaquine treatment closer to patients. The focus of this study was to assess the currently implemented test and treat strategy for patients with acute vivax malaria (as opposed to a strategy of mass test and treat.) In this context, we provide G6PD testing by community health workers as an alternative to be able to test and treat febrile patients in the community. Discussing potential implications for mass test and treat strategies is outside the scope of this work.

**Comment 5:** The results section is full of good data. The first paragraph of the discussion will benefit from a 500 word concise summary of important results from both quantitative and qualitative results.

**Reply:** We have added more details to the first paragraph of the discussion that summarizes the key findings from the study that will then be further discussed in the remainder of the discussion section. The addition now reads as follows (line 677-678, page 30), "*A lower percentage of females received radical cure compared to males, and children under 20 kilograms but over six months old were not tested and treated as per current policy*." We also added a sentence addressing one of Reviewer 2's comment from line 667 to 669 (page 30), "*Though our study focused on the Biosensor, findings relating to the test's implementation may be applicable to and relevant for other PoC G6PD tests that might be available and introduced later in Cambodia.*"

**Reviewer 2:**

**Comment 1:** First, a disclaimer: I am not familiar with methods for qualitative research and suggest that a specialist in this area should also evaluate the manuscript.

However, as a malariologist, I can say that the paper is very well written and addresses a question of major public health interest at times of tafenoquine introduction in vivax malaria treatment. Over more than five decades, primaquine has been deployed in Plasmodium vivax-endemic settings with no previous G6PD testing, but in Cambodia and other Southeast Asia the relatively high prevalence of severe G6PD deficiency variants in human populations is a major barrier to primaquine use – and surely to tafenoquine use in the near future.

**Reply:** Thank you for positive feedback and support regarding the relevance and importance of this research.

**Comment 2:** There are several findings in this study that are of interest for a broad audience of malariologists and public health specialists. For example, a community health worker (CHW) says: "(…) if we ask [patients] to come back to the village for the treatment [which is only available after testing], they will not come because they can't leave their personal belongings in the forest, [...], they will not come because they earn nothing yet." As a consequence, only 49.2% of eligible patients seen by CWHs reached health centers for G6PD testing. It remained unclear to me what happened to those patients who remained G6PD-untested? Were they treated with chloroquine alone for 3 days (either followed or not by weekly chloroquine) or left untreated? This is a key point, as the policy of requiring previous G6PD testing before providing radical vivax malaria treatment might actually prevent some patients from receiving any treatment!

**Reply 2:** Thank you for highlighting this important consideration. Individuals who are diagnosed with vivax malaria by community health workers in Cambodia are provided with schizontocidal treatment (ASMQ) before being referred to the health center for G6PD testing and primaquine treatment to make sure they are receiving at least schizontocidal treatment. We have clarified this in the "Overview of routine vivax case management policy in Cambodia" sub-section of the methods section (lines 197-198, page 8), "Routine G6PD testing was only introduced at health centers, hence patients diagnosed with vivax malaria by community health workers (CHWs) *were treated with schizontocidal drugs in the community but* required referral to a health center for testing prior to primaquine administration."

We also added clarification in the discussion section (lines 743-746, page 33), "Despite health center staff adopting the Biosensor at health center in over 90% of cases, access to G6PD testing and radical cure was still limited by significant challenges with referral from the community *where patients were diagnosed with vivax malaria and provided schizontocidal treatment*; less than half of eligible patients were successfully referred putting into question the overall feasibility and appropriateness of G6PD testing at the health center."

**Comment 3:** Overall, the discussion is well balanced. I particularly like the following comment: "Portrayal of primaquine [I would add: also of tafenoquine] as a cure or 'magic bullet' for vivax malaria could erode trust in both the treatment and healthcare providers". However, I miss comments about the barriers to the (future) implementation of tafenoquine in settings like Cambodia. Tafenoquine was the actual trigger of the renewed interest of diagnostic companies on developing point-of-care diagnostics for G6PD deficiency. If G6PD testing is not made widely available to all eligible patients, even those living in remote villages or deep in the forest, tafenoquine will never be successfully implemented in Cambodia.

**Reply:** Thank you for your thoughtful comment regarding the study finding's relevance to tafenoquine and appreciation of the 'magic bullet' discussion. We agree with the reviewer that findings are relevant for tafenoquine introduction as well. However, in Cambodia the use of ACTs is currently prohibiting considering tafenoquine introduction. To address this comment we have added the following to the discussion section (lines 779- 783, page 34-35): "*The availability of PoC G6PD diagnostics enables the consideration of higher more effective primaquine doses (67) as well as single dose tafenoquine (68). While the use of tafenoquine is not currently an option in Cambodia because of the drug's restriction for use with chloroquine only, ensuring adequate access to G6PD testing will be crucial for the broad roll out of these novel treatment options*."

**Comment 4**: I am a bit annoyed by the tendency to equate "point-of-care G6PD testing" with a particular product (STANDARD G6PD test, SD Biosensor), although several other relatively simple (and quantitative) diagnostic methods have been developed and can be used, if not by CHWs in remote villages, at least by technicians with very basic laboratory skills in small towns and cities. Relying on a single product, from a single manufacturer, to implement a countrywide policy of radical malaria cure seems too risky, given potential issues with timely procurement, distribution, and maintenance of the handheld devices and consumables. A comment on this topic might perhaps be appropriate, at the authors' discretion.

**Reply:** We have added a sentence addressing the applicability and relevance of our study findings to other potential G6PD test options to acknowledge that the Biosensor is one option, but the one that is currently being used. The sentence highlighted in italics was added to the beginning of the discussion from line 667 to 669 (page 30), "Cambodia's national malaria program has implemented G6PD testing using the Biosensor as part of vivax case management up to the health center level. *Though our study focused on the Biosensor, findings relating to the test's implementation may be applicable to and relevant for other PoC G6PD tests that might be available and introduced later in Cambodia*." Furthermore, how the test came to be chosen and implemented in Cambodia is mentioned in the introduction highlighting previous testing of the qualitative CareStart RDT.

Please let us know if you require any more clarification with regards to the amendments made. All authors have reviewed the revised manuscript and approved it for re-submission. Thank you very much for time.

Sincerely,

Sarah Cassidy-Seyoum
PhD Candidate and corresponding author
